# Preventive and therapeutic effects of *Nasturtium officinale* hydroalcoholic extract on cyclophosphamide-induced testicular toxicity in rats 

**DOI:** 10.22038/ajp.2025.25887

**Published:** 2025

**Authors:** Hamideh Aboutalebi, Fatemeh Alipour, Alireza Ebrahimzadeh-Bideskan

**Affiliations:** 1 * Department of* *Anatomy and* *Cell Biology, School of* *Medicine, Mashhad University of* *Medical Sciences, Mashhad, Iran*; 2 *Applied* *Biomedical* *Research Center, Mashhad University of Medical Sciences,* *Mashhad, Iran*

**Keywords:** Nasturtium officinale L, Cyclophosphamide, Sperm parameters, Testis, Rat

## Abstract

**Objective:**

Cyclophosphamide (Cy) as an alkylating chemotherapeutic agent with broad-spectrum efficacy in cancer treatment. Despite its wide spectrum of clinical usage, off-target multiple organ toxicity such as sperm and testicular injury is one of its toxic side effects. Since the* Nasturtium officinale* L. hydroalcoholic extract (NOE) contains a wide range of phytochemicals with various biological functions, the current study was designed to explore the protective potential of NOE on testicular toxicity caused by Cy in rats.

**Materials and Methods:**

Forty-eight adult male Wistar rats were randomly allocated into eight groups (n=6): control, Cy [received a single dose of 75 mg/kg, intraperitoneal (i.p)], NOE+Cy (Prevention): received NOE 500 and 1000 mg/kg/day, orally for 21 consecutive days and on the last day received Cy, Cy+NOE (Treatment): received NOE 500 and 1000 mg/kg/day, orally for 7 days after Cy administration for 21 consecutive days, and NOE (500 and 1000 mg/kg/day). After experiments, the testicular weight and volume, testosterone level, and sperm parameters as well as histologic and histomorphometric changes of testis were examined.

**Results:**

Base on the results, Cy caused significant decreases in testicular weight and volume, decreased testosterone level and reduced sperm count, and motility whereas increased sperm abnormality (p<0.05). Cy significantly reduced seminiferous tubules diameter, and height of the seminiferous epithelium (p<0.05). Furthermore, disorganization of seminiferous tubules diameter was increased in Cy group (p<0.05). Interestingly, pre and post-treatment with NOE could effectively improve testicular weight and volume, and testosterone level as well as sperm parameters. Furthermore, NOE administration ameliorated seminiferous tubules diameter diameter, seminiferous epithelium height (p<0.05).

**Conclusion:**

It is concluded that NOE may provide a potential protective effect for Cy-induced testicular damage.

## Introduction

Gonad toxicity is an important problem in chemotherapy of patients with various malignancies. A subsequent awkward situation that the cancer survivors may face, is subfertility or infertility (Blumenfeld 2012; Rouki et al. 2024). Cyclophosphamide (Cy), one of the most well-known alkylating chemotherapeutic agents, has been extensively utilized for the treatment of numerous malignancies (Hosseini et al. 2018; Parandin et al. 2023). Acrolein is known as the most toxic metabolite of Cy that has the capacity to induce harmful reactive oxygen species (ROS) (Jeelani et al. 2017). ROS have been considered a prominent contributory factor in the pathogenesis of infertility. ROS via damaging the sperm membrane, and sperm DNA, can affect fertility (Sun et al. 2015; Yang and Schaich 1996). Phosphoramide mustard another metabolite of Cy through introducing alkyl radicals, inhibits cell division through creating cross-links in DNA that is irreversible and eventually results in cell apoptosis (Patel et al. 1984; Zhang et al. 2005). Despite Cy wide spectrum of clinical uses, it can affect fertility in both sex (Fusco et al. 2021). Several studies have revealed that Cy is capable of producing a wide range of adverse impacts on male reproduction including, diminished sperm count, and motility, and increased abnormal sperm morphology (Hosseini et al. 2018). Moreover, decreased testicular weight as well as histological alterations of the testis has been reported following the treatment with Cy (Potnuri et al. 2018; Rezvanfar et al. 2008). Since sperm membrane contains high amount of polyunsaturated fatty acids, the male germ cells are exceedingly vulnerable to oxidative damage. Many reports have shown that ROS are associated with impaired sperm function and structure, including reduced motility, increased morphologically abnormal sperm, increase apoptosis in male germ cell, and subsequent hypo-spermatogenesis (Alahmar 2019). It seems that the administration of antioxidant agents might be the suitable medical strategies to lessen the toxicity induced by Cy. 

*Nasturtium officinale *L. R. Br. known as watercress, belongs to the Brassicaceae family, and it is a rich source of vitamins A, B, C, E as well as carotenoids, flavonoids, phenolics, glucosinolates, folic acid, sulfur compounds, proteins, and minerals (Akbari Bazm et al. 2019; Sathasivam et al. 2022). Based on previous *in vitro* and *in vivo* studies, *Nasturtium officinale *hydroalcoholic extract (NOE) possesses antioxidant, anti-inflammatory, anti-hyperlipidemic, hepatoprotective and, nephroprotective properties (Karami et al. 2018; Sedaghattalab et al. 2021). Supplementation with NOE has been reported to exert testiculo-protective effect against oxymetholone-induced testicular toxicity (Akbari Bazm et al. 2019). 

The aim of the current study was to determine the testiculo- protective role of NOE on sperm parameters and testicular tissue after Cy damage.

## Materials and Methods

The current study was conducted in accordance with the National Institutes of Health Guide for the Care and Use of Laboratory Animals (Publications No. 80-23, revised 1978) and approved by Research Ethics Committee at Mashhad University of Medical Sciences, Mashhad, Iran (Ethical Approval Code: IR.MUMS.MEDICAL.REC.1397.499 (.

### Preparation of the extract

The plant was harvested manually from the rivers around North Khorasan Province, Iran in late spring (2022). An herbarium specimen voucher (No FUMH-E1008) was deposited in the Herbarium Ferdowsi University of Mashhad, Iran. In brief, at first, the leaves of the plant were washed and dried in shade. Then, the samples were ground into fine powder and placed in an oven for three days at 39°C and then, the extraction was done through percolation procedure in 70% ethanol at room temperature (24 ± 2°C). The resulting solution was filtered and ethanol was evaporated at 40°C; the extract was then stored at −20°C before use (Natanzi et al. 2009; Rad et al. 2021).

### Animals

In this study, 48 adult male Wistar rats (weighing 180±20 gr) were obtained from laboratory animal house of Mashhad University of Medical Sciences and kept under controlled conditions (22–24^o^C and a relative humidity of 50–55% with a 12 hr dark-light cycle) with free access to food and drinking water *ad libitum*. After one-week adaptation, the animals were randomly divided into eight groups (n=6): Control group received no treatment. 

Cy group received a single intraperitoneal (i.p.) injection of Cy (75 mg/kg) on the first day of the experiment (Aboutalebi et al. 2022).

NOE 500+Cy group (preventive) received 500 mg/kg NOE for 21 consecutive days orally and on the last day received a single i.p. injection of Cy (Ibrahim et al. 2015) and a week later on the 28^th^ day animals were sacrificed .

NOE 1000+Cy group (preventive) received 1000 mg/kg extract for 21 consecutive days and on the last day, received a single i.p. injection of Cy, and a week later on the 28^th^ day animals were sacrificed (Al-Snafi 2020).

Cy+ NOE 500 group (Treatment): seven days after a single dose of Cy (75 mg/kg), 500 mg/kg extract was given orally for 21 consecutive days. 

Cy+ NOE 1000 group (Treatment): seven days after a single dose of Cy (75 mg/kg), 1000 mg/kg extract was given orally for 21 consecutive days.

NOE 500 group received only 500 mg/kg/day extract orally for 21 consecutive days.

NOE 1000 group received only 1000 mg/kg/day extract orally for 21 consecutive days.

### Hormone assay

After the experiments period, the animals were weighed and deeply anesthetized using an i.p injection of 90 mg/kg of ketamine hydrochloride and 10 mg/kg of xylazine. Then, the blood samples were taken through cardiac puncture to measure serum level of testosterone by radioimmunoassay.

### Testis volume

Testes were removed and the total volume was estimated by immersion method. Briefly, the testis was placed carefully into a graduated cylinder with certain amount of normal saline. Then, the testicular volume was measured by subtracting the final and initial volume of normal saline (Parhizkar et al. 2014).

### Sperm parameters assays

For this purpose, the epididymis was separated and minced to 1 ml preheated Hanks' Balanced Salt Solution (HBSS) solution, then, incubated in CO_2 _incubator at 37^o^C (15 min) to swim out of sperm. Sperm suspensions were analyzed to assess sperm parameters including sperm count, motility, and morphology.

### Sperm count

One drop (10 μl) of diluted sperm suspension was transferred into a Neubauer Haemocytometer and the number of sperm was counted at a magnification of 40 x. The number of spermatozoa was calculated in 4 different squares and then, the total number of spermatozoa is reported in millions/ml (10^6^). Sperm count was performed in duplicate by an independent skilled technician to the study.

### Sperm motility

One drop of sperm suspension was transferred on a microscopic slide and coverslip. The number of sperm with progressive movement was evaluated in 10 microscopic fields at magnification of 100 x and expressed as the percentage of motile sperm.

### Sperm morphology

One drop of sperm suspension from each animal was smeared onto glass slides then, fixed in 96% ethanol and allowed to air dry. Sperm smears were then stained by Papanicolaou staining. In brief, after washing in distilled water (3 min), the samples were stained with hematoxylin staining solution (5 min). Following the washing, the samples were placed into 1% acid alcohol and then, into 96% alcohol I and II (15 sec), respectively. After that, the samples were stained with orange G6 (OG6) staining solution (5 min) followed by 96% alcohol I and II (15 s) and then stained with eosin azure 50 (EA 50) staining solution (5 min), followed by 96 % alcohol I and II (15 sec) and finally, 100% alcohol (1 min). To evaluate the sperm morphology, a total of 200 spermatozoa were assessed using light microscope at ×100 magnification.

### Histopathologic evaluation

 For this purpose, the right testis from all the animals was fixed in 10% formaldehyde, and then, processed according to the standard paraffin embedding protocol and finally, the tissue blocks were sectioned at a thickness of 5 µm. The sections were then deparaffinized, rehydrated and stained with hematoxylin and eosin (H&E). Ten stained sections from each testis were evaluated using an Olympus microscope (BX51, Japan) for quality structural changes. 

### Histomorphometric evaluation

 The diameter of 20 transversely cut seminiferous tubule per testis was determined. In the same tubules, the seminiferous epithelium height from the basement membrane to the lumen of the epithelium was measured at two different regions at 20 × magnification.

### Statistical analysis

Data was analyzed using SPSS (version 16) software. To compare the parametric data (testicular weight, volume, testosterone concentration, histomorphometric data, and sperm parameters), ANOVA and Tukey's tests were used. Furthermore, to compare the Non-parametric data (histopathological evaluation) Kruskal-Wallis and Mann-Whitney tests were used. To determine the normal distribution of data, Kolmogorov-Smirnov test was used. p<0.05 was considered the significance level. Values are expressed as Mean ± SD. 

## Results

### Hormone analysis

**Figure 1:**
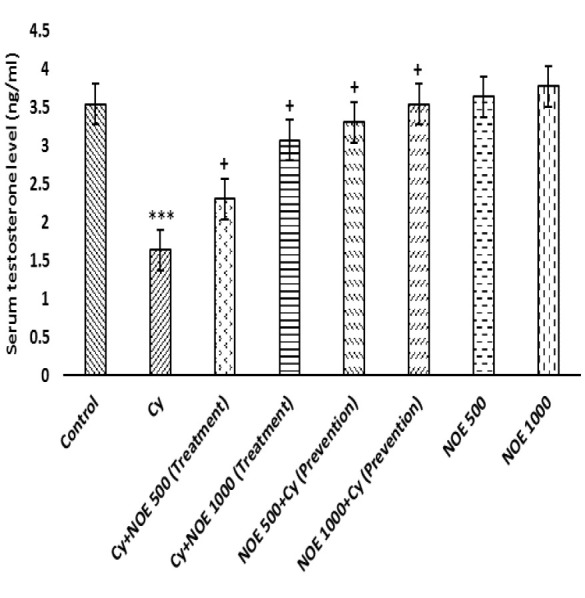
Comparison of the mean of serum testosterone level after treatment with NOE. Data is presented as Mean ± SD. ***p<0.001 compared to control; +p<0.05 compared to NOE 500 + Cy (Prevention); NOE 1000 + Cy (Prevention); Cy + NOE 500 (Treatment); Cy+ NOE 1000 (Treatment).

The results showed that Cy significantly decreased testosterone level in comparison with the control group (p<0.001) while administration of NOE at dose of 500 and 1000 mg/kg in both preventive and therapeutic groups could significantly increase testosterone level (p<0.05). However, treatment with high dose of NOE (preventive and therapeutic groups) was more effective than low dose. There was not any difference in testosterone level of NOE groups in comparison with the control group (Figure.1). 

### Body weight and testis weight

Cy treatment resulted in lower body weight compared to the control group. However, this reduction was not significant. An increase was found in body weight of NOE -treated groups (preventive and therapeutic groups) compared to the Cy group though; this increase was not statistically significant. There was no difference in the body weight of NOE groups compared to the control group. The weight of the testes significantly decreased in Cy group compared to control group (p<0.001) whereas in NOE -treated groups (preventive and therapeutic groups) a significant increase was observed compared to the Cy group (p<0.001). No significant difference was observed in testicular weight between the low and high dose of NOE -treated groups. There was no significant difference in testicular weight of NOE groups in comparison with the control group (Table 1).

### Testis volume

Cy-treated rats had significant reductions in testicular volume compared to the control group (p<0.001) and administration of NOE at doses of 500 and 1000 (preventive and therapeutic groups) significantly improved the testicular volume compared to the Cy group (p<0.001). No significant difference was observed in testicular volume between the low and high dose of NOE (Table 1).

### Sperm parameters

Table 2 summarizes the results obtained from the sperm assessments. The total sperm counts in Cy group declined significantly compared to the control group (p<0.001) while significant restoration of the same was observed in the rats treated with 500 and 1000 dose of NOE compared to the Cy group (p<0.001). NOE at dose of 1000 mg/kg was more effective than 500 mg/kg in improving the sperm count. In addition, no significant difference was found in either preventive and therapeutic groups that received high dose (1000 mg/kg) of NOE. No significant difference in sperm counts was observed between the NOE groups and control group (Table 2). Based on the results, there was a significant decrease in the mean percentage of forward sperm motility in the Cy group compared with all other treatment groups (p<0.001). Conversely, the percentage of forward motility was significantly higher in the NOE-treated groups (500 and 1000 mg/kg) in both preventive and therapeutic groups compared to the Cy group (p<0.001). However, NOE at the dose of 1000 mg/kg was more effective than 500 mg/kg. There was no significant difference in sperm motility between the NOE groups and control group (Table 2). The percentage of abnormal sperm morphology was significantly increased in the Cy-treated group compared to the other groups (p<0.001), whereas this parameter was significantly lower in the NOE-treated groups at two doses than in the Cy group (p<0.001). No significant difference was observed between NOE-treated groups (500 and 1000 mg/kg) in both preventive and therapeutic groups (Table 2). 

### Histopathological findings

**Figure 2:**
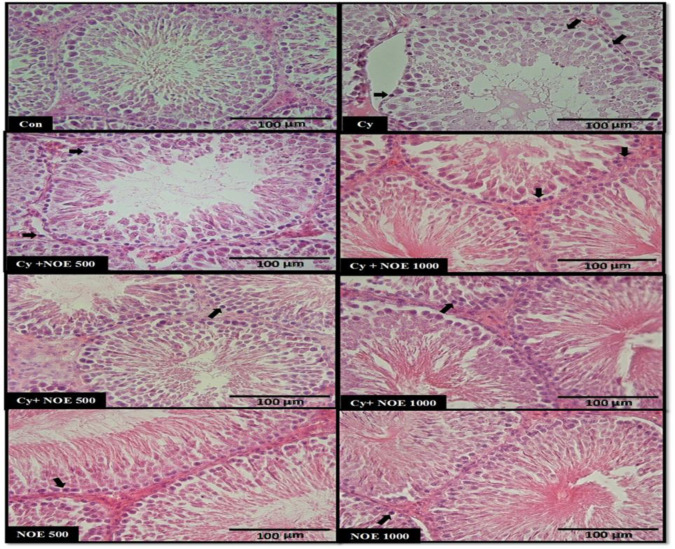
Photographs of several seminiferous tubules in rats stained with H&E (x 40). Testicular microscopic images of control (Con) group, cyclophosphamide (Cy) group, NOE (Nasturtium officinale L. extract) 500 + Cy (Prevention), NOE 1000 + Cy (Prevention), Cy + NOE 500 (Treatment), and Cy + NOE 1000 (Treatment). Note the presence of pyknotic nuclei with arrows. Scale bar = 100 µm.

Based on the results, Cy caused many adverse histological changes in testicular tissue including germinal epithelium disorganization, and detachment of spermatogenic cells, and degenerative cells in interstitial tissues as well as picnotic nuclei were observed. Conversely, the testis of rats treated with NOE (preventive and therapeutic groups) displayed mild degenerative tissue changes with less structural changes in spermatogenic cells, lower sloughed germ cell into the lumen compared to the Cy group. Furthermore, tubular sections in NOE groups exhibited normal feature as shown in control group (Figure 2).

### Histomorphometric findings

The morphometric examination showed normal testicular structure in control group. In Cytreated group, the diameter and height of seminiferous tubules were less than the control group (p<0.001). Conversely, NOE (500 and 1000 mg/kg) in both preventive and therapeutic groups significantly increased the mean diameter and height of seminiferous epithelium compared to Cytreated rats (p<0.001), (Figures 3a and 3b). 

## Discussion

Cancer chemotherapy has been shown to exert both temporary and permanent detrimental impacts on male fertility (Ghafouri-Fard et al. 2021). Cy as a common anti-neoplastic agent possesses a broad spectrum of antitumor activity in the treatment of various human cancers. Despite its pharmacological benefits, it possesses devastating effects on male reproduction (Hosseini et al. 2018). Fertility preservation strategies in patients undergoing chemotherapy can be considered as important parallel treatment in reducing the side effects of chemotherapy (Lambertini et al. 2016). Based on the findings of recent studies, some medicinal plants have shown significant improvement in the androgen status and fertility index after chemotherapy (Qu et al. 2020). 

As our data showed, Cy caused low testosterone concentration which is consistent with other studies (Hamzeh et al. 2019). Cy may cause substantial reduction in the activity of enzymes involved in testosterone biosynthesis, which may be due to the oxidative damage and alterations in the gene expression pattern of Leydig cells caused by Cy (Bakhtiary et al. 2016). Our results showed that NOE improved testicular endocrine functions by increasing testosterone levels. It has been previously reported that apigenin, a phenolic compound of NOE, interferes with the production of testosterone and modulates the secretion of this hormone (Li et al. 2011). Hussein et al. (2013) reported that another member of Brassicaceae family (*Eruca Sativa *L.) also possess androgenic activity or can trigger testicular steroid synthesis, improve spermatogenesis, and scavenge free radicals (Hussein 2013). Our data also showed that Cy caused a reduction in the relative body weigh but the reduction was not significant. Some previous studies have indicated that Cy decreases the body weight (Kamiya et al. 2021). Cy probably through influence on the amount of fat mass through low appetite and preventing the protein synthesis, reduces body weight. However, Razak et al. (2019) showed no significant change in body weight of Cy-treated group (Razak et al. 2019). The contradictory results obtained on the adverse effects of Cy on body weight in various studies are probably resulted from the different doses, different routes of administration, different duration of treatment as well as the sensitivity of the animals used. 

Additionally, in our study, Cy caused a significant decrease in testicular weight and volume as compared to the control group. The mentioned result can be explained by a decrease in the number of germ cells, a notable decrease in spermatogenesis rate and atrophy of Leydig cells (Rezvanfar et al. 2008) (Onaolapo et al. 2018). In our study, NOE could increase testis weight and volume compared to Cy-treated group. This finding can be ascribed on the one hand, to the antioxidant properties of NOE in averting atrophy, necrosis, and testis degeneration and on the other hand, through increasing the number of germ cells and stimulating the spermatogenesis, on testicular weight and volume (Akbari Bazm et al. 2019). Testicular volume and weight are regarded as good clinical markers for male fertility (Bhushan et al. 2016). Based on previous studies, *Eruca sativa* L, another member of Brassicaceae family has testiculo-protective effect against toxic compounds, and could enhance the volume and weight of this organ which is consistent with our study (Abd El-Aziz et al. 2016). 

Furthermore, in our study, Cy caused a significant decrease in sperm count and motility, and increased the number of abnormal sperm. These findings are likely resulted from the oxidative effects of Cy, as sperm is highly sensitive to oxidative stress (OS) (Ghafouri-Fard et al. 2021). The cessation of spermatogenesis and the reduction of germ cells number are in line with the reduction in sperm count caused by Cy (Yuan et al. 2014). In accordance with our findings, Elgazar et al. (2016) showed that male patients exposed to Cy had reduced total sperm count and lack of spermatogenic cycles (Elgazar 2016). Intriguingly, administration of NOE increased significantly sperm count, motility, and reduced abnormal sperm. Since testosterone is required for spermatogenesis, the increase in sperm count is likely a result of increased testosterone levels with NOE treatment. This result is in accordance with those observed by Akbari Bazm et al. (2019) (Akbari Bazm et al. 2019). One of the probable mechanisms of NOE effects on increasing sperm parameters is due to its antioxidant properties. Since OS reduces the enzymatic or non-enzymatic antioxidants in Leydig cells, it can affect the process of spermatogenesis by decreasing the synthesis and secretion of testosterone, resulting in reduced sperm count (Darbandi et al. 2018). Furthermore, decreased sperm motility in our study may be resulted from the devastating effect of Cy on the flagellum. Since ATP is considered the primary energy source for sperm motility, exposure to Cy may result in detrimental changes in energy metabolism and subsequent loss of sperm motility (Vernet et al. 2004). Thus, reduced sperm motility might be resulted from the impaired sperm flagellar function or impaired ATP regeneration as a result of ROS generation (Razak et al. 2019) and reduced Na/K ATPase activity can change the membrane lipid peroxidation of sperm (Woo et al. 2000). Enhanced sperm motility following NOE treatment seems to depend on low production of ROS. Also, Cy caused a significant decrease in the seminiferous tubules diameter and seminiferous epithelium height as well. In contrast, NOE administration could prevent more epithelial destruction (Rigi Manesh et al. 2018). Due to the fact that the effective compounds of herbal extracts have a remarkable superiority over chemical drugs; they may act as an effective tool to treat infertility problems (Jaradat and Zaid 2019). We suggest that antioxidants components in NOE are able to reduce harmful effects of Cy on testis.

The results revealed that NOE could improve sperm parameters and maintain the important morphometric indicators such as seminiferous tubules diameter and seminiferous epithelium height near to normal. It seems NOE because of antioxidant properties, can be a potential testiculo-protective agent against multiple Cy- induced testicular toxicities. Further studies are needed to use NOE as an antioxidant supplement during chemotherapy with Cy.

**Table 1:** Body and testicular weight and volume of rats treated with Cy and/or NOE

**Groups **	**Body weight (g) **	**Testis weight (mg) **	**Volume (mm3)**
Control	288.25± 2.06	1.55± 0.17	1.27± 0.12
Cy	245± 24.27	0.67±0.03^***^	0.47± 0.10^***^
Cy+ NOE 500 (Prevention)	276.75±12.14	1.47±0.15^###^	0.975±0.17^###^
Cy+ NOE 1000 (Prevention)	254.25±26.8	1.52±0.17^&&&^	1.3±0.081^&&&^
Cy+ NOE 500 (Treatment)	262.5±16.42	1.15±0.20^¥¥¥ ^	0.97±0.17^¥¥¥^
Cy+ NOE 1000 (Treatment)	268.25± 17.5	1.25±0.12^€€€^	1.1± 0.11^€€€^
NOE 500	279.5±20.59	1.65±0.1	1.25±0.12
NOE 1000	293.75±6.29	1.7±0.2	1.32±0.09

Data is presented as Mean ± SD. *= significant different from Cy group (p<0.001); #= significant different from NOE 500 + Cy (Prevention) (p<0.001); &= significant different from NOE 1000 + Cy (Prevention) (p<0.001); ¥= significant different from Cy + NOE 500 (Treatment) (p<0.001); €= significant different from Ext 1000 + Cy (Prevention) (p<0.001). Cy: cyclophosphamide; NOE: Nasturtium officinale L. extract.

**Table 2:** Sperm analysis of rats treated with Cy and/or NOE.

**Groups **	**Sperm count (×10** ^6^ **) **	**Sperm motility (%) **	**Normal morphology (%)**
Control	262.17±24	78± 6.48	88.25± 4.1
Cy	145.82±47^***^	32.75±3.0^***^	38.5± 3.5^***^
Cy+ NOE 500 (Prevention)	242±26^###^	71± 11.74^###^	70.75± 4.85^###^
Cy+ NOE 1000 (Prevention)	253.29±28^&&&^	74.25± 7.5^&&&^	79.75± 9.7^&&&^
Cy+ NOE 500 (Treatment)	186.35±57^¥¥¥ ^	68.25± 3^¥¥¥ ^	69.75± 4.2^¥¥¥^
Cy+ NOE 1000 (Treatment)	241.94±40.20^€€€^	73± 7.87^€€€ ^	72.75± 5.67^€€€^
NOE 500	257.11± 31	77.75±4.4	84.25± 2.75
NOE 1000	263.05±34	79.75± 2.9	92± 4.24

Data is presented as Mean±SD. *= significant different from Cy group (p<0.001); #= significant different from NOE 500+ Cy (Prevention) (p<0.001); &= significant different from NOE 1000 + Cy (Prevention) (p<0.001). ¥= significant different from Cy+ NOE 500; €= significant different from Cy + NOE 1000 (Treatment) (p<0.001).

**Figure 3a:**
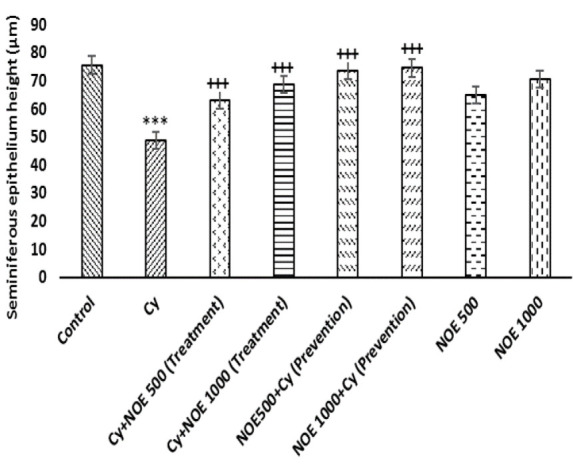
Comparison the mean of seminiferous epithelial height after treatment with NOE. Data is presented as Mean±SD. ***p<0.001 compared with control; +++p<0.001 compared with NOE 500 + Cy (Prevention); NOE 1000 + Cy (Prevention); Cy + NOE 500 (Treatment); Cy + NOE 1000 (Treatment).

**Figure 3b:**
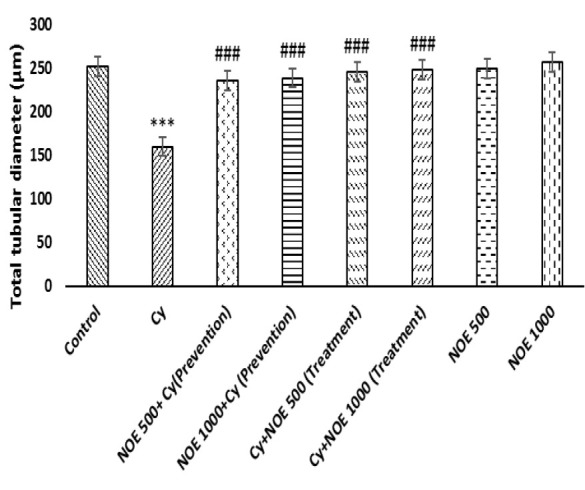
Comparison the mean of total tubular diameter after treatment with NOE. Data is presented as Mean±SD. ***p<0.001 compared with control; ###p<0.001 compared with NOE 500 + Cy (Prevention); NOE 1000+ Cy (Prevention); Cy + NOE 500 (Treatment); Cy + NOE 1000 (Treatment).
